# Targeted protein degradation using deGradFP in *Trypanosoma brucei*

**DOI:** 10.12688/wellcomeopenres.17964.2

**Published:** 2022-10-19

**Authors:** Midori Ishii, Bungo Akiyoshi

**Affiliations:** 1 Department of Biochemistry, University of Oxford, Oxford, OX1 3QU, UK

**Keywords:** Trypanosoma brucei, targeted protein degradation, deGradFP, degron, kinetoplastid, kinetochore

## Abstract

Targeted protein degradation is an invaluable tool in studying the function of proteins. Such a tool was not available in *Trypanosoma brucei*, an evolutionarily divergent eukaryote that causes human African trypanosomiasis. Here, we have adapted deGradFP (degrade green fluorescent protein [GFP]), a protein degradation system based on the SCF E3 ubiquitin ligase complex and anti-GFP nanobody, in *T. brucei*. As a proof of principle, we targeted a kinetoplastid kinetochore protein (KKT3) that constitutively localizes at kinetochores in the nucleus. Induction of deGradFP in a cell line that had both alleles of KKT3 tagged with yellow fluorescent protein (YFP) caused a more severe growth defect than RNAi in procyclic (insect form) cells. deGradFP also worked on a cytoplasmic protein (COPII subunit, SEC31). Given the ease in making GFP fusion cell lines in *T. brucei*, deGradFP can serve as a powerful tool to rapidly deplete proteins of interest, especially those with low turnover rates.

## Introduction

Kinetoplastids are a group of unicellular flagellated eukaryotes found in diverse environmental conditions ( d’Avila-Levy *et al.*, 2015). They belong to the phylum Euglenozoa (Discoba/Excavata) and are evolutionarily distant from commonly studied model eukaryotes such as yeasts, worms, flies, and humans (Opisthokonta) ( Cavalier-Smith, 2010; Keeling & Burki, 2019). Understanding their biology could therefore provide insights into the extent of conservation/divergence among eukaryotes and lead to a deeper understanding of biological systems and evolution of eukaryotes. Importantly, three neglected tropical diseases are caused by parasitic kinetoplastids: African trypanosomiasis, Chagas disease, and leishmaniasis ( Horn, 2022; Rao *et al.*, 2019). Human African trypanosomiasis (sleeping sickness) is caused by *Trypanosoma brucei*, which also causes the cattle disease, nagana, that leads to weight loss and anemia in livestock and imposes a huge burden on economic development in affected regions. Understanding the biology of kinetoplastids could facilitate the design of new drugs against kinetoplastid parasites.

Inducible depletion of a target protein is an essential tool in biology ( Prozzillo *et al.*, 2020). In *Trypanosoma brucei*, this can be achieved by RNAi ( Alsford *et al.*, 2011; Ngô *et al.*, 1998) and Tet-off system ( Merritt & Stuart, 2013) at the RNA level, as well as by conditional knockout at the gene level using Cre-LoxP ( Kim *et al.*, 2013). Although powerful in many cases, these approaches are not efficient in reducing the level of proteins that have slow turnover rates. Targeted degradation tools could circumvent this problem and have been used in other organisms ( Damerow *et al.*, 2015; Madeira da Silva *et al.*, 2009; Nabet *et al.*, 2018; Nishimura *et al.*, 2009; Uhlmann *et al.*, 2000; Wheeler *et al.*, 2015). However, such tools were not available in *Trypanosoma brucei*, to our knowledge.

 In this study, we have adapted the deGradFP (degrade green fluorescent protein) system which was originally established in *Drosophila melanogaster* ( Caussinus *et al.*, 2011). It relies on the expression of VhhGFP4 fused with a truncated F-box protein. VhhGFP4 is an anti-GFP nanobody that recognizes GFP and some derivatives such as yellow fluorescent proteins (YFP) and Venus ( Saerens *et al.*, 2005), while an F-box protein is a substrate-recognition subunit of the SKP1–CUL1–F-box (SCF) E3 ubiquitin ligase complex that catalyzes the ubiquitylation of target proteins ( Petroski & Deshaies, 2005). In deGradFP, a substrate-recognition domain of an F-box protein is replaced by VhhGFP4 so that GFP-fusion proteins are ubiquitylated by the SCF complex, leading to their degradation via the 26S proteasome pathway ( Caussinus & Affolter, 2016). deGradFP or modified versions have been used in mammalian cells, *Caenorhabditis elegans*, zebrafish, and plants ( Shin *et al.*, 2015; Sorge *et al.*, 2021; Wang *et al.*, 2017; Yamaguchi *et al.*, 2019). Here, we show that deGradFP successfully depletes a kinetochore protein and a COPII subunit in the procyclic form of *T. brucei* cells.

## Results

**Figure 1.**
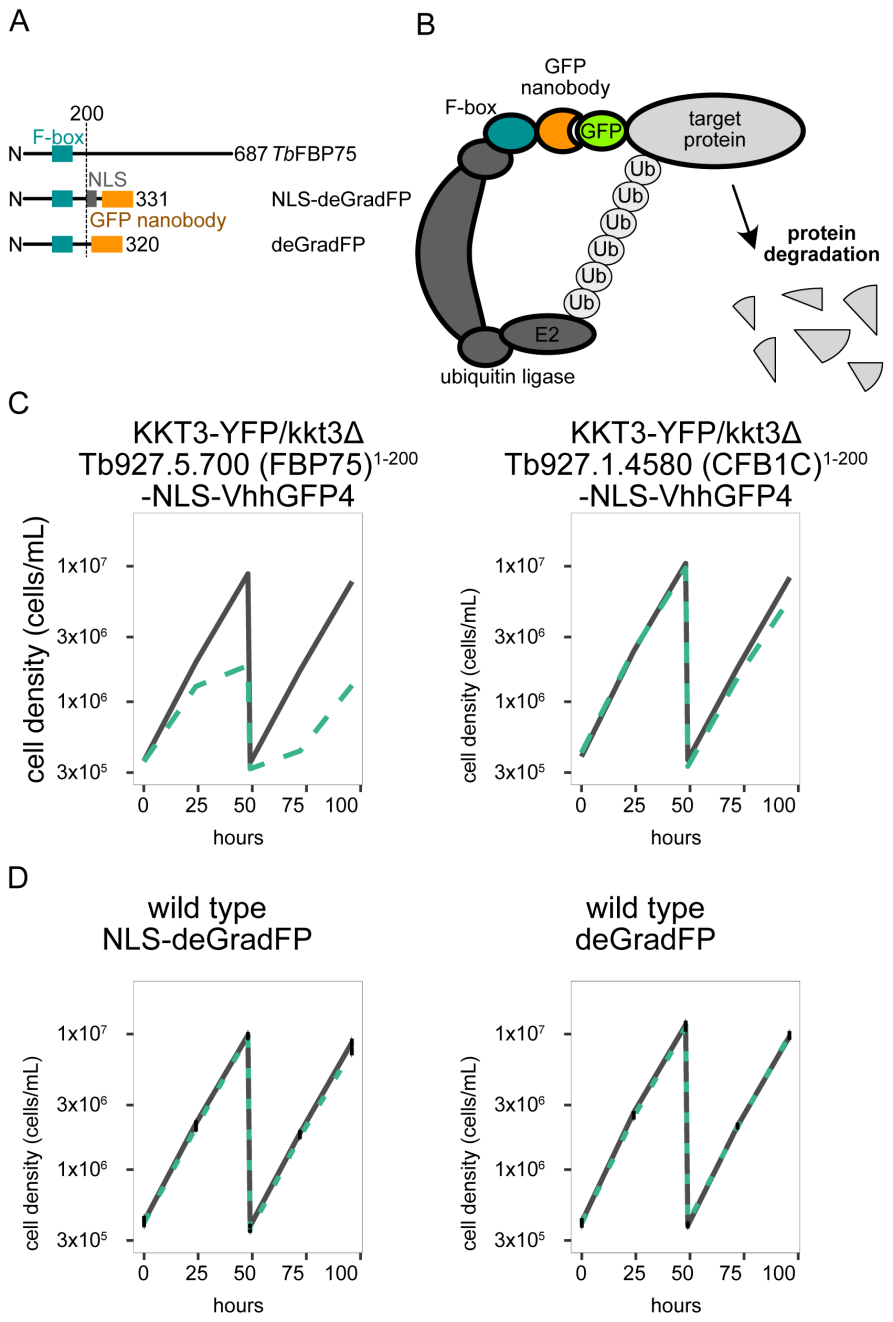
deGradFP in *Trypanosoma brucei*. ( **A**) Schematic of *Tb*FBP75 (Tb927.5.700), NLS-deGradFP, and deGradFP, highlighting the putative F-box domain, NLS, and GFP nanobody (vhhGFP4). ( **B**) deGradFP forms a complex with an endogenous ubiquitin ligase complex which transfers ubiquitins to the target protein tagged with GFP. Ubiquitylated target proteins are then degraded by the 26S proteasome. ( **C**) Growth curve for KKT3-YFP/kkt3∆ with induction of Tb927.5.700 (FBP75) ^1-200^-NLS-VhhGFP4 (left) and Tb927.1.4580 (CFB1C) ^1-200^-NLS-VhhGFP4 (right). N=2. Representative growth curves are shown. Cell line: BAP2398, BAP2399 ( **D**) Growth curve for wild-type procyclic cells with NLS-deGradFP (left) or deGradFP (right). N=3. Error bars are SEM. Cell line: BAP2395, BAP2511. For all growth curves, deGradFP was induced with 1 μg/ml doxycycline and cultures were diluted at day 2. Gray lines are uninduced controls. Green dashed lines are doxycycline-treated cells. N=3. Error bars are SEM. Cell line: BAP2395, BAP2511.

To establish a targeted protein degradation method in *T. brucei*, we chose a protein that has a slow turnover rate. KKT3 is a kinetochore protein that constitutively localizes at kinetochores and does not show any obvious fluctuation in its abundance during the cell cycle, implying that it is a stable protein ( Akiyoshi & Gull, 2014). In fact, the half-life of KKT3 has been estimated to be much longer than transiently-localized kinetochore proteins ( Tinti *et al.*, 2019). To apply deGradFP in *T. brucei*, we made a construct that expresses an F-box domain fused with a nuclear localization signal (NLS) and the anti-GFP nanobody VhhGFP4 ( Saerens *et al.*, 2005) ( Figure 1A, B). The fusion protein was expressed from a derivative of pDEX777 that integrates at the 177 bp repeats on minichromosomes and allows doxycycline-inducible expression ( Kelly *et al.*, 2007; Nerusheva & Akiyoshi, 2016). Putative F-boxes of Tb927.5.700 and Tb927.1.4580 (called cyclin-like F-box protein 1C, CFB1C ( Benz & Clayton, 2007)) were tested. When expressed in cells that have KKT3-YFP as the sole copy of KKT3 (KKT3-YFP/kkt3∆) ( Figure 1C), the construct containing Tb927.5.700 caused growth defects, implying that expression of Tb927.5.700 ^1-200^-NLS-VhhGFP4 led to degradation of KKT3-YFP. In contrast, expression of CFB1C ^1-200^-NLS-VhhGFP4 did not cause strong growth defects. This means that expression of VhhGFP4 (at least when fused to the F-box of CFB1C) was not sufficient to disrupt the function of KKT3-YFP. We therefore used the F-box from Tb927.5.700 (named FBP75 herein for F-box protein 75 kDa) for subsequent experiments. Besides the construct with an NLS to target nuclear proteins (pBA2675: NLS-deGradFP), we also made another one without an NLS to target cytoplasmic proteins (pBA2705: deGradFP). Induction of these deGradFP systems in wild-type procyclic cells with 1 µg/mL doxycycline did not cause any growth defect ( Figure 1D), showing that expression of deGradFP itself does not cause growth defects.

**Figure 2.**
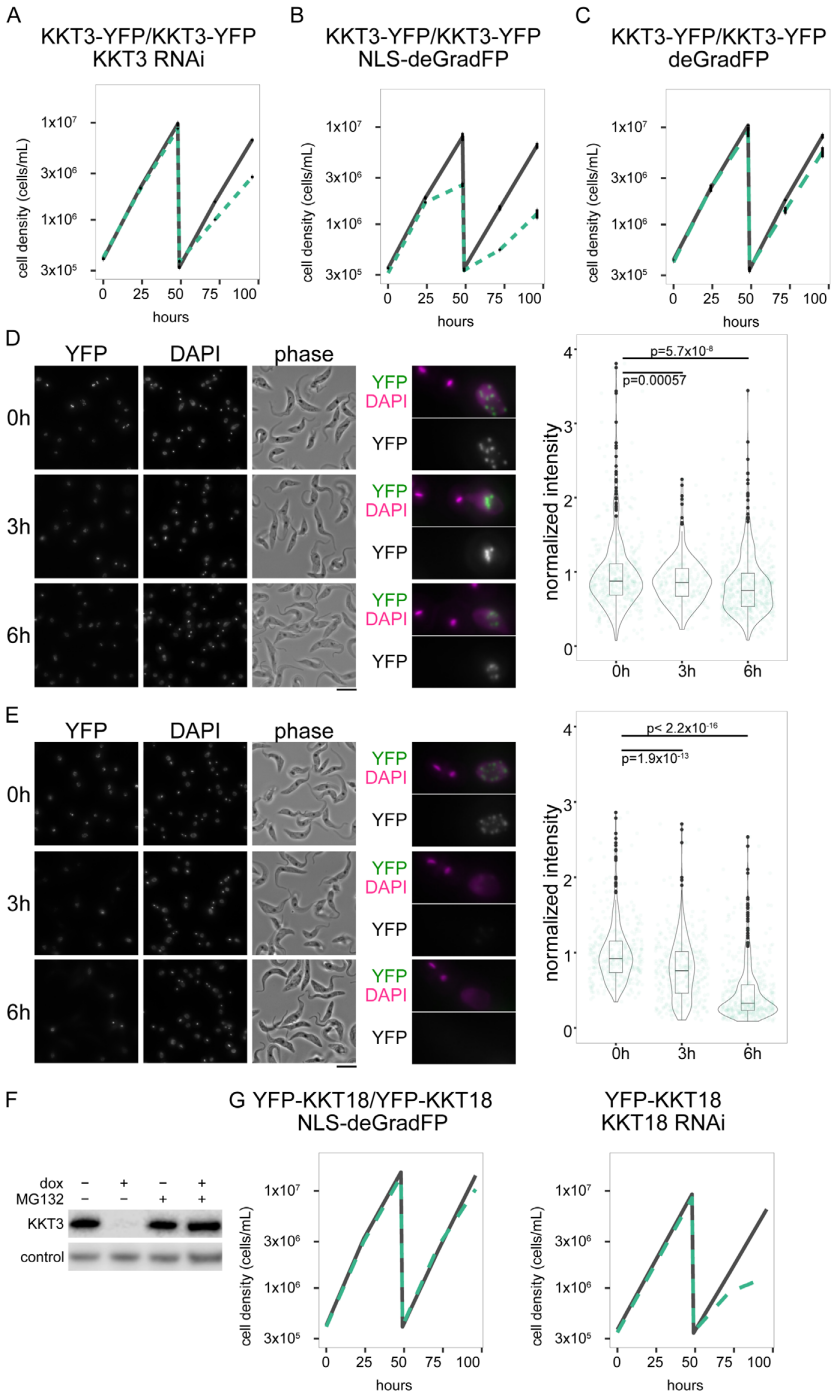
deGradFP depletes a nuclear protein KKT3 more efficiently than RNAi. ( **A**, **B**, **C**) Growth curves for KKT3 knockdown by RNAi ( **A**: N=4, cell line: BAP2512), KKT3-YFP depletion by deGradFP with NLS ( **B**: N=3, cell line: BAP2513) and KKT3-YFP with deGradFP without NLS ( **C**: N=3, cell line: BAP2514). Error bars are SEM. ( **D**, **E**) Images of KKT3 knockdown by RNAi ( **D**: cell line: BAP2512), and KKT3-YFP depletion by deGradFP with NLS ( **E**: cell line: BAP2513). (Left) Examples of cells at 0 h, 3 h, and 6 h after induction. YFP and DAPI images are maximum intensity projection. Bars, 10 µm. (Centre) Examples of cells from left images. (Right) Plot of total YFP signal inside the nucleus (>239 cells in each condition). Data were normalized with the mean value at 0 h. P-values were calculated by Welch two sample t-test. ( **F**) KKT3-YFP cells with NLS-deGradFP were incubated with either 1 µg/mL doxycycline or 10 µM MG132, or both for 6 hours. Control is untreated cells. KKT3-YFP was detected by immunoblot using anti-GFP antibody. PFR2 was used as a loading control. Cell line: BAP2513. ( **G**) Growth curve for YFP-KKT18 with NLS-deGradFP (left) and KKT18 RNAi (right). Representative growth curves are shown. N=2 (NLS-deGradFP) and 3 (RNAi). Cell lines: BAP2593, BAP2122. For all growth curves, RNAi or deGradFP was induced with 1 μg/ml doxycycline and cultures were diluted at day 2. Gray lines are uninduced controls. Green dashed lines are doxycycline-treated cells.

 We next used a cell line in which both alleles of KKT3 were C-terminally tagged with YFP using a PCR-based method in one transfection step ( Dean *et al.*, 2015). We found that induction of NLS-deGradFP in this cell line caused more severe growth defects than RNAi ( Figure 2A and B) ( Marcianò *et al.*, 2021). We did not observe severe growth defects with deGradFP without NLS in KKT3-YFP cells, showing the importance of NLS to target nuclear proteins in our system ( Figure 2C). Microscopy analysis confirmed that NLS-deGradFP caused more significant depletion of KKT3 at six hours than RNAi ( Figure 2D and E). The fact that induction of NLS-deGradFP in wild-type cells did not cause any growth defect ( Figure 1D) means that the observed growth defect was due to specific degradation of YFP-tagged KKT3. In the deGradFP system, YFP-tagged target proteins ubiquitylated by the SCF ubiquitin ligase complex are degraded by the proteasome system ( Caussinus & Affolter, 2016). Consistent with this possibility, addition of the proteasome inhibitor MG132 prevented degradation of KKT3-YFP ( Figure 2F), suggesting that our deGradFP system in *T. brucei* relies on the proteasome-dependent protein degradation pathway as expected. We note that NLS-deGradFP did not work well for another kinetochore protein KKT18 ( Figure 2G). In contrast, RNAi-mediated depletion of KKT18 caused growth defects and reduction of protein.

**Figure 3.**
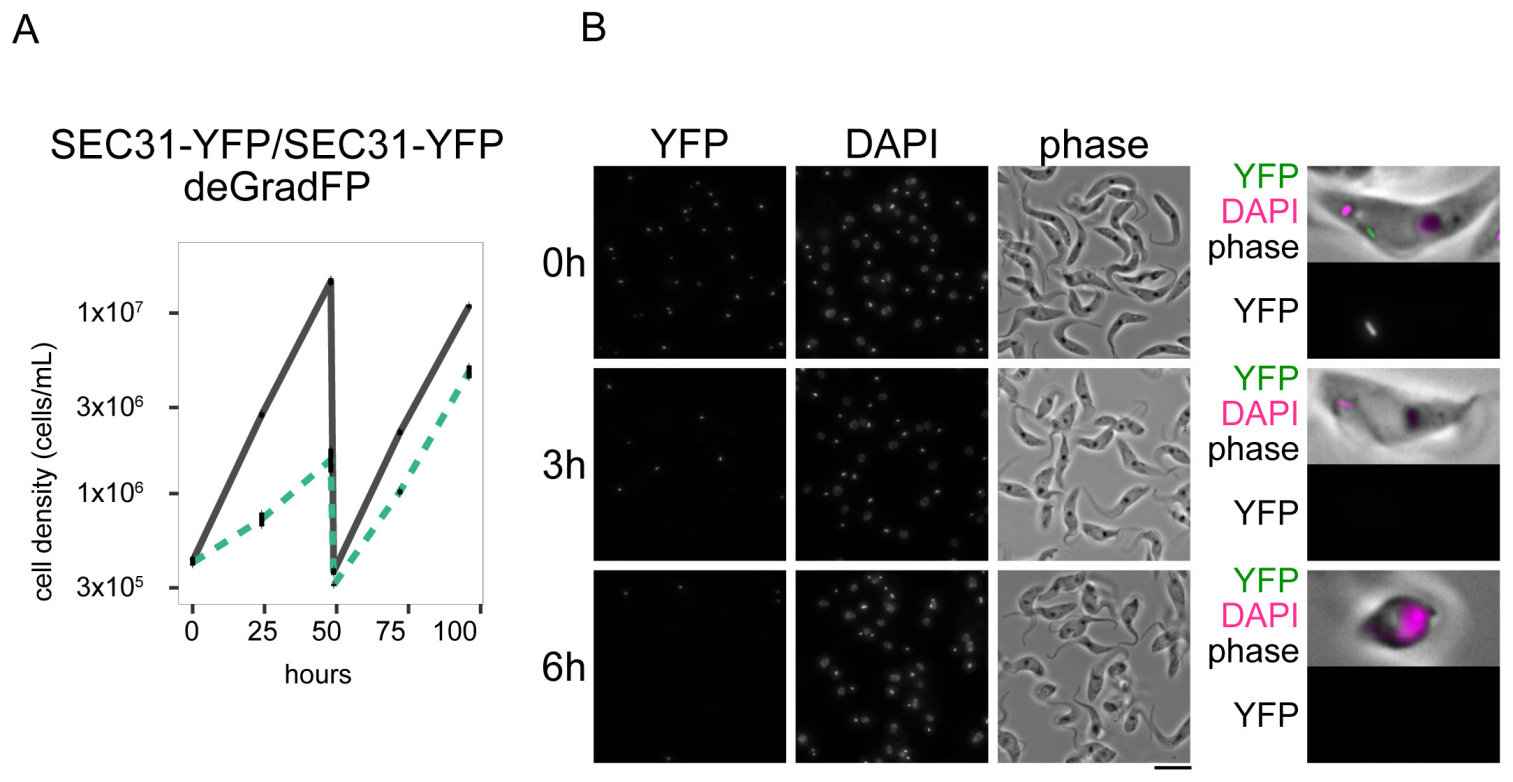
Depletion of a cytoplasmic protein SEC31 by deGradFP. ( **A**) Growth curve for SEC31-YFP with deGradFP. N=4. Error bars are SEM. ( **B**) Examples of cells at 0 h, 3 h, and 6 h after induction. YFP and DAPI images are maximum intensity projection. Bar, 10 µm. Cell line: BAP2518. For all growth curves, RNAi or deGradFP was induced with 1 μg/ml doxycycline and cultures were diluted at day 2. Gray lines are uninduced controls. Green dashed lines are doxycycline-treated cells.

 We next targeted a cytoplasmic protein SEC31 using a deGradFP construct that lacks an NLS. SEC31 is a subunit of COPII and localizes at the endoplasmic reticulum (ER) exit site ( Hu *et al.*, 2016). Both alleles of SEC31 were C-terminally tagged in a CRISPR cell line ( Beneke *et al.*, 2017). Induction of deGradFP caused a strong growth defect ( Figure 3A and B), which is apparently more severe than RNAi-mediated depletion of SEC31 reported in a previous study ( Hu *et al.*, 2016). These results therefore show that deGradFP can efficiently deplete both nuclear and cytoplasmic proteins in *T. brucei*.

## Discussion

In *T. brucei*, it is easy to tag genes at the endogenous locus using plasmid- or PCR-based methods ( Beneke *et al.*, 2017; Dean *et al.*, 2015; Kelly *et al.*, 2007; Kovářová *et al.*, 2022). Taking advantage of the inducible expression system ( Poon *et al.*, 2012; Wirtz & Clayton, 1995), we have shown that deGradFP can induce targeted protein degradation in *T. brucei*. The depletion kinetics is faster than the RNAi-mediated depletion method, at least for KKT3. Our results therefore show that deGradFP can be a powerful tool in characterizing depletion phenotypes in *T. brucei*. It is, however, important to note that deGradFP has some limitations. For example, it has been suggested that deGradFP does not work if GFP is not accessible ( Caussinus *et al.*, 2011; Caussinus & Affolter, 2016). It is also essential that target proteins have lysines that can be ubiquitylated by deGradFP. Furthermore, it is critical that GFP-fusion proteins retain enough functionality to support cell growth because the deGradFP system utilizes the VhhGFP4 nanobody that recognizes GFP or its derivatives. If necessary, this system could be modified to use nanobodies against other epitope tags or even the protein of interest itself to induce degradation of the target ( Aguilar *et al*., 2019).

The function of the F-box protein used in this study (FBP75) remains unknown. We also do not know which SKP1 or cullin proteins interact with FBP75 and whether those proteins are expressed in other life stages. It therefore remains unknown whether FBP75-based deGradFP works in bloodstream form cells. If it does not work, other F-box proteins could be utilized to deplete proteins of interest in bloodstream form cells ( Benz & Clayton, 2007; Rojas *et al.*, 2017). In any case, it is our hope that deGradFP will prove to be a useful protein degradation tool to facilitate studies of *Trypanosoma brucei*.

## Methods

### Plasmids

All plasmids used in this study are listed in Table 1. To make pBA2675 (inducible expression of FBP75 ^1–200^-NLS-VhhGFP4: NLS-deGradFP for nuclear proteins) and pBA2676 (inducible expression of CFB1C ^1–200^-NLS-VhhGFP4), synthetic DNA BAG181 (pBA2675) and BAG182 (pBA2676) (GeneArt, Thermo Fisher) were digested with HindIII/BamHI and subcloned into pBA310 cut with the same enzymes. The NLS sequence was derived from the La protein ( Marchetti *et al.*, 2000). To make pBA2705 (Inducible expression of FBP75 ^1–200^-VhhGFP4: deGradFP for cytoplasmic proteins), NLS was removed from pBA2675 by PCR with BA3647/BA3648. 12.5 µL of 2x PrimeSTAR MAX (Takara), 1 µL of 3 ng/µL pBA2675 plasmid, 1 µL each of 10 µM forward and reverse primers, and 11 µL of MilliQ water were mixed (30 cycles of 98°C 10 s, 55°C 15 s, 72°C 35 s). The PCR reaction was incubated with 1 µL of DpnI (NEB) at 37°C for 1 hr and was transformed into NEB 5-alpha competent *E. coli* (NEB). To make pBA871 (hairpin RNAi targeting 49–518 bp of KKT18 coding sequence) and pBA1061 (hairpin RNAi targeting 2562–3072 bp of the KKT3 coding sequence), synthetic DNA BAG34 (KKT18) and BAG55 (KKT3) (GeneArt, Thermo Fisher) were digested with HindIII/BamHI and subcloned into pBA310 cut with the same enzymes.

**Table 1.** Plasmids used in this study.

Name	Description
pPOTv7 (eYFP, Hyg)	PCR-based eYFP-tagging vector, hygromycin marker ( Dean *et al.*, 2015)
pPOTv7 (eYFP, G418)	PCR-based eYFP-tagging vector, G418 marker ( Dean *et al.*, 2015)
pBA101	YFP-KKT18 tagging at endogenous KKT18 locus, hygromycin ( Akiyoshi & Gull, 2014)
pBA871	Inducible expression of KKT18 hairpin RNAi (targeting 49–518 bp of KKT18 coding sequence), integrate at 177 bp, phleomycin marker
pBA310	Inducible expression vector, integrate at 177 bp, phleomycin marker ( Nerusheva & Akiyoshi, 2016)
pBA1061	Inducible expression of KKT3 hairpin RNAi (targeting 2562–3072 bp of KKT3 coding sequence), integrate at 177 bp, phleomycin marker
pBA2675	Inducible expression of FBP75 ^1–200^-NLS-VhhGFP4, integrate at 177 bp, phleomycin marker (NLS-deGradFP for nuclear proteins)
pBA2676	Inducible expression of CFB1C ^1–200^-NLS-VhhGFP4, integrate at 177 bp, phleomycin marker
pBA2705	Inducible expression of FBP75 ^1–200^-VhhGFP4, integrate at 177 bp, phleomycin marker (deGradFP for cytoplasmic proteins)

### Trypanosome cells

All cell lines used in this study were derived from the TREU 927 procyclic form cells and are listed in Table 2. SmOxP927 expresses Tet repressor and T7 RNA polymerase ( Poon *et al.*, 2012), while PCF 1339 expresses Tet repressor, T7 RNA polymerase, and the Cas9 nuclease ( Beneke *et al.*, 2017). Cells were grown at 28°C in SDM-79 medium (Life Technologies, Thermo Fisher) supplemented with 10% heat-inactivated fetal calf serum (Sigma) and 7.5 µg/mL hemin, as well as puromycin (Sigma) and appropriate drugs ( Brun & Schönenberger, 1979).

**Table 2.** Trypanosome cell lines used in this study.

Name	Description
SmOxP927	TREU927 procyclic cells that expresses TetR and T7 RNAP ( Poon *et al.*, 2012)
PCF 1339	TREU927 procyclic cells that expresses TetR, T7 RNAP, and Cas9 ( Alves *et al.*, 2020)
BAP167	YFP-KKT18 ( Akiyoshi & Gull, 2014)
BAP2122	YFP-KKT18, KKT18 RNAi
BAP1123	KKT3-YFP/kkt3∆ ( Nerusheva *et al.*, 2019)
BAP2398	KKT3-YFP/kkt3∆, FBP75 ^1–200^-NLS-VhhGFP4
BAP2399	KKT3-YFP/kkt3∆, CFB1C ^1–200^-NLS-VhhGFP4
BAP2395	FBP75 ^1–200^-NLS-VhhGFP4
BAP2511	FBP75 ^1–200^-VhhGFP4
BAP2464	KKT3-YFP/KKT3-YFP
BAP2512	KKT3-YFP/KKT3-YFP, KKT3 RNAi
BAP2513	KKT3-YFP/KKT3-YFP, FBP75 ^1–200^-NLS-VhhGFP4
BAP2514	KKT3-YFP/KKT3-YFP, FBP75 ^1–200^-VhhGFP4
BAP2466	SEC31-YFP/SEC31-YFP
BAP2518	SEC31-YFP/SEC31-YFP, FBP75 ^1–200^-VhhGFP4
BAP2567	YFP-KKT18/YFP-KKT18
BAP2593	YFP-KKT18/YFP-KKT18, FBP75 ^1–200^-NLS-VhhGFP4

 To make the homozygous KKT3-YFP cell line, two YFP-tagging cassettes were amplified from pPOTv7 (YFP, Hyg) or pPOTv7 (YFP, G418) ( Dean *et al.*, 2015) by PCR using BA1821/BA1822 ( Table 3). 25 µL of 2x PrimeSTAR MAX (Takara), 1 µL of 30 ng/µL template pPOT plasmid, 1 µL each of 10 µM forward and reverse primers, and 22 µL of MilliQ water were mixed (30 cycles of 98°C 10 s, 55°C 15 s, 72°C 1 min). 50 µL of PCR products were transfected into SmOxP927 ( Poon *et al.*, 2012) by electroporation. Transfected cells were selected by addition of 50 μg/mL hygromycin (Sigma) and 30 μg/mL G418 (Sigma) and cloned by dispensing dilutions into 96-well plates. Clones were screened by diagnostic PCR of genomic DNA using BA523/BA2352.

**Table 3.** Sequence of primers and synthetic DNA used in this study.

Name	Description
BA3647	CACCTGCTCCACCGTCCTCCATGTGCGGCA
BA3648	GGAGGACGGTGGAGCAGGTGGAGCAGGTGT
BA1821	GTAATGGAGTTTGTGAGGTGCTTGATGAGGAAAAATTCCCCCTTTCGGAGGAACTCAACCAGATGCTCTACGGTGGCGTGGGTTCTGGTAGTGGTTCC
BA1822	GAAATGCGACAGCAGACGGAAACGGAAAAAAAAATAAAAAAAAAGAGAGGGCTATCTGTAATTCTTTACGTACATCACTTCCAATTTGAGAGACCTGTGC
BA523	TATGTCTGTTTATTGCCCAC
BA2352	GATCGATC GCGGCCGC TTTTCAGTTGCTATAGGCCT
BA3633	GCTCAAGGGAAATGTGGAAAGAGCTCGCCACTAAGCACTTTTCGGCAATTCAACATATTAATAACCTCAAGTTTCTGCAGGGTTCTGGTAGTGGTTCCGG
BA3634	ATATATGCAACCCGGCGACAAACAAACACCGCACAGGTGCAAAGGCACACAAACATGTTTTCCTTTGAGTGCCATGTGTGCCAATTTGAGAGACCTGTGC
BA3635	GAAATTAATACGACTCACTATAGGGGCGTGGCTCGCTCACGAGCGTTTTAGAGCTAGAAATAGC
G00 (BA2931)	AAAAGCACCGACTCGGTGCCACTTTTTCAAGTTGATAACGGACTAGCCTTATTTTAACTTGCTATT TCTAGCTCTAAAAC
BA3638	TTGTTTAGGATCACAACGCT
BA3639	GTACAGACACGTCCGTACAA
BA3376	CACTTTATCCTTGGGAGGGGTTTGCCAACCGCGTGCCTGTGGTCTTCAGCCGTGCATTTCTTTCCTCTTTCTTCAGCCTCGTATAATGCAGACCTGCTGC
BA3377	ACCTCTACATCAGAAAGGGGAAACACACAAGCGCGCACCTCGTCCGTTGGAAGCTGCAACAACTCTTTTACGCAGGTCATACTACCCGATCCTGATCCAG
BA3378	GAAATTAATACGACTCACTATAGGATCGTTGTAGGGCGCTGACCGTTTTAGAGCTAGAAATAGC
BA385	GATCGATC GCGGCCGC ATGATTGGCTGTTATGCAAC
BA384	GATCGATC GCGGCCGC GCTGGCCCTCTTTACATGAA
BAG34	GATCGATCGATC AAGCTT GCTTGTGTGTTTCCCCTTTCTGATGTAGAGGTTACTTTAGTCCTCTGTGAATTGGGAAAACTTCTGCAGAGATGGCTTTACGCCACGCGCTACAAGTTGG TTTCCTCATTCGAGGTATTTCTTCATTTATTTGTGCACGTAGCGGACAGAGAACCGAAAAGGATAGCCAACGCTTTCCTCGGTTCATGTAAAGAGGGCCA GCACCTGTCGTTGCTGGCAGATGTATGTGAAACCCTCTTCTCACCATCCCGGATTTCCCTTCTTGCTGAAGTGGACGTAGAGCTTTTCTTAACATTCTTA AAGTTCCTCTGTGACATTCCTGTATTGAAGGATCGGCTCGGTAATGTTCTGGTGAAGATTCTCCTGGAATTCCTCTCCGCTGCGGTGGAGCGCGATGGGG ACTACCGTCAACCCCGAGCATGTGCAAGCGTGCTCATCACCCTCACACGTGGGTCAAAGGCAAACAAAGAAAAGGCGGACCCTCATTTCTAAGTACGGTC AGGTGTCGTAGCACTGCATTGAATTCGATTGCCATTCTCCGAGTGTTTTAGCGTGACGGCCGCAGGGGTCCCATAATCTTTGTTTGCCTTTGACCCACGT GTGAGGGTGATGAGCACGCTTGCACATGCTCGGGGTTGACGGTAGTCCCCATCGCGCTCCACCGCAGCGGAGAGGAATTCCAGGAGAATCTTCACCAGAA CATTACCGAGCCGATCCTTCAATACAGGAATGTCACAGAGGAACTTTAAGAATGTTAAGAAAAGCTCTACGTCCACTTCAGCAAGAAGGGAAATCCGGGA TGGTGAGAAGAGGGTTTCACATACATCTGCCAGCAACGACAGGTGCTGGCCCTCTTTACATGAACCGAGGAAAGCGTTGGCTATCCTTTTCGGTTCTCTG TCCGCTACGTGCACAAATAAATGAAGAAATACCTCGAATGAGGAAACCAACTTGTAGCGCGTGGCGTAAAGCCATCTCTGCAGAAGTTTTCCCAATTCAC AGAGGACTAAAGTAACCTCTACATCAGAAAGGGGAAACACACAAGC GGATCC GATCGATCGATC ( KKT18 hairpin)
BAG55	GATCGATCGATC AAGCTT ACGTCACATGCTGCTTAACGGTGATTGGATACGCTACTACCACTTTTATCCTATGGAGGAAGAAGGAGGCGACTCAGTCGCTGTCACATATCATATTCAG CCGGGACGTACTGGTGTTACATTTTTCAACCATAGTTTTTCTGTGCACTCAGCTGTGCTGTCAGTGTTGGAACACATCGTATACGTCGTAGATCGTGTTG ATATCGAGGAAGATAATGACGTGGCGCGTATCTTGTCGTTGGCACAAGCATTGAATGAGGAGAAGAAGATCTACGATGTCCTTCAATTGGTCGAAACCCA CGACACACATATGTTAAAACAGCGGCGGTCTCCCGGTATTATGTCTGTTTATTGCCCACCACAAACAGCATTTCAATGCAATGGTGATCCCTTTGTATTT GTTCGCTGGTACCGCTTCCATATGGAAAACTCTATGAGTGGCTTTATGCTCTCCAACGGGGCTGTGCAGGTGTTTGTAGGCGGGAAATACGAGTTACGGT GGCTGGATGACAAAGGCGGACCCTCATTTCTAAGTACGGTCAGGTGTCGTAGCACTGCATTGAATTCGATTGCCATTCTCCGAGTGTTTTAGCGTGACGG CCGCAGGGGTCCCATAAGTCATCCAGCCACCGTAACTCGTATTTCCCGCCTACAAACACCTGCACAGCCCCGTTGGAGAGCATAAAGCCACTCATAGAGT TTTCCATATGGAAGCGGTACCAGCGAACAAATACAAAGGGATCACCATTGCATTGAAATGCTGTTTGTGGTGGGCAATAAACAGACATAATACCGGGAGA CCGCCGCTGTTTTAACATATGTGTGTCGTGGGTTTCGACCAATTGAAGGACATCGTAGATCTTCTTCTCCTCATTCAATGCTTGTGCCAACGACAAGATA CGCGCCACGTCATTATCTTCCTCGATATCAACACGATCTACGACGTATACGATGTGTTCCAACACTGACAGCACAGCTGAGTGCACAGAAAAACTATGGT TGAAAAATGTAACACCAGTACGTCCCGGCTGAATATGATATGTGACAGCGACTGAGTCGCCTCCTTCTTCCTCCATAGGATAAAAGTGGTAGTAGCGTATCCA ATCACCGTTAAGCAGCATGTGACGT GGATCC GATCGATCGATC ( KKT3 hairpin)
BAG181	GATCGATC AAGCTT ATGGGTGGTGGAGCAGCGGTGTCGTCTGGTGACGACAGCGCCGCGGCATCTTCGGGTAACGACACCACCACAACGGATGGTAATCATGGCGGGAGGTACG CCTGGGAATGCGTTGACGACGTCGGGGGCGCTTTCAACGGTTCCATGAATGGCAACACCTCCCTGCCAAAAGGTTCAAACATCTGTGAGACTCACGGGTG TTGGAGTAAGCAAATACTCCGAAGGGACATTTGCTGCGAGCGGAGCTACGTTCACCCTGTCAGCGCTGCTTGGCGTTGCAGCAGCGTTCTTTCACTTCCA GTGTCACTCCTTGACGAGGTGTTCACTTTTTTGCATCCCGAGGATCTCTGCAGGGTATTGGAAGTATGCCGGTTTTTCTTCTCTGCCGCTGTAAGGTCCG ATCGTACCGCCTGGAGGTCCGTATGCCTTTCGCTATGGAAAAACAAGCAGGGGCTCTCGCGCGTGGTGCGTGAATGGCCGTCTGTGGAGGAGGTATGCCG ACAGGAAGACTTGGAGTCGATTTGTGTGCAACAAGCGTTTGCACATGAATATTTCAGCTGGGGAACCATTAACAACATGGTGCCGCACATGGAGGACGGT ACCGGTCGAGGACACAAGCGGTCACGTGAACAA GGAGCAGGTGGAGCAGGT GTCCAACTGGTGGAGTCTGGTGGCGCTTTGGTGCAGCCAGGTGGCTCTCTGCGTTTGTCCTGTGCCGCTTCTGGCTTCCCAGTGAACCGCTATTCCATGC GCTGGTATCGCCAGGCTCCAGGCAAAGAGCGTGAGTGGGTAGCCGGTATGTCCAGCGCGGGTGATCGTAGCTCCTATGAAGACTCCGTGAAGGGCCGTTT CACCATCAGCCGTGACGATGCCCGTAACACGGTGTATCTGCAAATGAACAGCTTGAAACCTGAAGATACGGCCGTGTATTACTGTAATGTGAACGTGGGCTTC GAGTATTGGGGCCAAGGCACCCAGGTCACCGTCTCCAGCTAA GGATCC GATCGATC ( FBP75's first 600 bp, NLS, GlyAlaGly linker x2, VhhGFP4)
BAG182	GATCGATC AAGCTT ATGTTTTTGAAGGAGGGAAACAGTGAAATGCGGTTGAGCGGAGCTACGTTCTTGAAGGGCGCTGTTGATGATTTCAATGCATGTGAGTCCCCTGTTTTAT CATCCGGAAGCAGTTTGGTTCTCACAAGTCCTGCTCTGAGCACCTCAGACACGTGCCGATCTGCGATGCGTGAAACCATCCCTTCCGTTTGTGCTGGCGC AGGCCTCAGCAGTCGCTGCTCAACTGATTGCACTGTGCTGAAGTGTGTGAACCCGCTGCATGTTAAGTTCCAGGAGCCGTCAGGGTTGTCAGGGGCTGAT TCAGTGGACTCGTTTGACTCGTTGGGTGACCTTTCCGAGTTGCATGAGGAACATCAACATGTTTCTTCTGTTGCCTCTGAAAAGCCTTTGACCCTTCACG ATGTGCCGGAATCCGTCCTGTATGGTGCCATTCCATACCTTTCCCTTCCAGACGTCATTGCCTTATCACGGACATGTAAGAAGTTTCACAAACTGGTGCA GGGATACTTTGCAGTGAATGAGCATGGCGTGATGAGCATCCCCGCCTTTGACACCCGCAGCTTCATGCAGTACCGTCCAGAGAGGAAACCTCCGGTAACG ACCGGTCGAGGACACAAGCGGTCACGTGAACAA GGAGCAGGTGGAGCAGGT GTCCAACTGGTGGAGTCTGGTGGCGCTTTGGTGCAGCCAGGTGGCTCTCTGCGTTTGTCCTGTGCCGCTTCTGGCTTCCCAGTGAACCGCTATTCCATGC GCTGGTATCGCCAGGCTCCAGGCAAAGAGCGTGAGTGGGTAGCCGGTATGTCCAGCGCGGGTGATCGTAGCTCCTATGAAGACTCCGTGAAGGGCCGTTT CACCATCAGCCGTGACGATGCCCGTAACACGGTGTATCTGCAAATGAACAGCTTGAAACCTGAAGATACGGCCGTGTATTACTGTAATGTGAACGTGGGC TTCGAGTATTGGGGCCAAGGCACCCAGGTCACCGTCTCCAGCTAA GGATCC GATCGATC

 To make the homozygous SEC31-YFP and YFP-KKT18 cell lines, donor DNA templates were amplified from pPOTv7 (YFP, Hyg) or pPOTv7 (YFP, G418) ( Dean *et al.*, 2015) with BA3633/BA3634 (SEC31) or BA3376/3377 (KKT18). 25 µL of 2x PrimeSTAR MAX (Takara), 1 µL of 30 ng/µL template pPOT plasmid, 1 µL each of 10 µM forward and reverse primers, and 22 µL of MilliQ water were mixed (30 cycles of 98°C 10 s, 55°C 15 s, 72°C 1 min). sgRNA template amplified with BA3635/G00 (SEC31) and BA3378/G00 (KKT18) using 12.5 µL of 2x PrimeSTAR MAX (Takara), 4 µL of 10 µM G00 primer, 4 µL of 10 µM target specific forward primer, 4.5 µL MilliQ water (35 cycles of 98°C 10 s, 60°C 30 s, 72°C 15 s). All the PCR products were mixed and purified using a QIAquick PCR purification kit (QIAGEN), eluted with 50 µL of MilliQ water, and then co-transfected into PCF 1339 ( Alves *et al.*, 2020; Beneke *et al.*, 2017) by electroporation. Transfected cells were selected by addition of 50 μg/mL hygromycin (Sigma) and 30 μg/mL G418 (Sigma) and cloned by dispensing dilutions into 96-well plates. Clones were screened by diagnostic PCR of genomic DNA using BA3638/BA3639 (SEC31) and BA385/BA384 (KKT18). 5 µg of RNAi and deGradFP constructs were linearized by NotI-HF (NEB) and transfected into YFP-tagged cell lines (3 clones each for KKT3-YFP, SEC31-YFP, and YFP-KKT18) or SmOxP927 by electroporation and selected by addition of 5 µg/mL phleomycin (Sigma). For induction of deGradFP or RNAi, doxycycline (Sigma) was added to the medium to a final concentration of 1 μg/mL. Cell growth was monitored using a CASY cell counter (Roche) and plotted with ggplot in R.

### Fluorescence microscopy

Cells were harvested by centrifugation at 800 g for 5 min, washed in PBS, settled onto glass slides for 5 min, and fixed with 4% paraformaldehyde for 5 min. Following three washes in PBS (5 min each), cells were permeabilized with 0.1% NP-40 in PBS for 5 min, washed three times in PBS (5 min each), and embedded in mounting media (1% 1,4-Diazabicyclo [2.2.2]octane (DABCO), 90% glycerol, and 50 mM sodium phosphate pH 8.0) containing 100 ng/mL DAPI. Images were captured at room temperature on a Zeiss Axioimager.Z2 microscope (Zeiss) installed with ZEN using a Hamamatsu ORCA-Flash4.0 camera with 63x objective lenses (1.40 NA). 22 optical slices spaced 0.24 μm apart were collected. Images were processed in ImageJ/Fiji ( Schneider *et al.*, 2012). Maximum intensity projection images were generated by Fiji software ( Schneider *et al.*, 2012). Total YFP intensity in the nucleus was measured using 3D Objects Counter with default settings in Fiji as follows. DAPI images were first used to segment the nucleus by removing regions that have top 0.2% intensity (that correspond to kDNA signals) and then by selecting objects that have the size of nuclei (5.3–40 µm ^3^). YFP total intensity inside the nucleus was measured using a redirect function in 3D Objects Counter. 3D Plots were made with ggplot in R.

### Immunoblots

Cells were harvested by centrifugation (800 g, 5 min) and washed with 1 ml PBS. Then cells were resuspended in 2× LDS sample buffer (Thermo Fisher) with 0.1 M DTT. Denaturation of proteins was performed for 5 min at 95°C. SDS-PAGE and immunoblots were performed by standard methods using the following antibodies: anti-GFP (OriGene, rabbit TP401, 1:2000) for YFP-tagged proteins and L8C4 (anti-PFR2, 1:1500) ( Kohl *et al.*, 1999) for a loading control. Bands were visualized by horseradish-peroxidase-conjugated sheep anti-mouse-IgG antibodies (GE Healthcare) on an ODYSSEY Fc Imaging System (LI-COR).

## Data Availability

Figshare: Extended Data for "Targeted protein degradation using deGradFP in *Trypanosoma brucei*", https://doi.org/10.6084/m9.figshare.19960244 ( Ishii & Akiyoshi, 2022) This project contains the following underlying data: - Fig1_1.csv (raw data for growth curve of NLS-deGradFP in wild-type cells, BAP2395) - Fig1_2.csv (raw data for growth curve of deGradFP in wild-type cells, BAP2511) - Fig1_3.csv (raw data for growth curve of NLS-deGradFP in KKT3-YFP/kkt3∆, BAP2398) - Fig1_4.csv (raw data for growth curve of Tb927.1.4580 ^1-200^-NLS-VhhGFP4 in KKT3-YFP/kkt3∆, BAP2399) - Fig2_1.csv (raw data for growth curve of KKT3-YFP/KKT3-YFP with KKT3 RNAi, BAP2512) - Fig2_2.csv (raw data for YFP intensity of KKT3-YFP/KKT3-YFP with KKT3 RNAi, BAP2512) - Fig2_3.czi (raw microscopy image, 0h control, KKT3-YFP/KKT3-YFP with KKT3 RNAi, BAP2512) - Fig2_4.czi (raw microscopy image, 3h, KKT3-YFP/KKT3-YFP with KKT3 RNAi, BAP2512) - Fig2_5.czi (raw microscopy image, 6h, KKT3-YFP/KKT3-YFP with KKT3 RNAi, BAP2512) - Fig2_6.csv (raw data for growth curve of KKT3-YFP/KKT3-YFP with NLS-deGradFP, BAP2513) - Fig2_7.csv (raw data for YFP intensity of KKT3-YFP/KKT3-YFP with NLS-deGradFP, BAP2513) - Fig2_8.czi (raw microscopy image, 0h control, KKT3-YFP/KKT3-YFP with NLS-deGradFP, BAP2513) - Fig2_9.czi (raw microscopy image, 3h, KKT3-YFP/KKT3-YFP with NLS-deGradFP, BAP2513) - Fig2_10.czi (raw microscopy image, 6h, KKT3-YFP/KKT3-YFP with NLS-deGradFP, BAP2513) - Fig2_11.csv (raw data for growth curve of KKT3-YFP/KKT3-YFP with deGradFP, BAP2514) - Fig2_12.csv (raw data for growth curve of YFP-KKT18 with KKT18 RNAi, BAP2122) - Fig2_13.csv (raw data for growth curve of YFP-KKT18 with NLS-deGradFP, BAP2593) - Fig2_14.tif (raw immunoblot data for KKT3-YFP, GFP antibody) - Fig2_15.tif (raw immunoblot data for PFR2 loading control, L8C4 antibody) - Fig3_1.csv (raw data for growth curve of SEC31-YFP/SEC31-YFP with deGradFP, BAP2518) - Fig3_2.csv (raw microscopy image, 0h control, SEC31-YFP/SEC31-YFP with deGradFP, BAP2518) - Fig3_3.csv (raw microscopy image, 3h, SEC31-YFP/SEC31-YFP with deGradFP, BAP2518) - Fig3_4.csv (raw microscopy image, 6h, SEC31-YFP/SEC31-YFP with deGradFP, BAP2518) - Table3.csv (Sequence for primers, synthetic DNA, and plasmids) Data are available under the terms of the Creative Commons Zero “No rights reserved” data waiver (CC0 1.0 Public domain dedication).
