# Supplementary material for: Targeted protein degradation using deGradFP in Trypanosoma brucei
Source: Wellcome Open Res. 2022 Oct 19;7:175. Originally published 2022 Jun 23. [Version 2] doi: 10.12688/wellcomeopenres.17964.2 (PMC9277568; doi:10.12688/wellcomeopenres.17964.2)
Supplement: Peer review history

# Peer review: Reviewer response for version 1

**DOI:** 10.21956/wellcomeopenres.19908.r51225

**Published:** 2022-07-11

**Authors:** David Horn

**Affiliations:** 1 The Wellcome Trust Centre for Anti-Infectives Research, School of Life Sciences, University of Dundee, Dundee, UK

Approaches for conditional expression of specific mRNAs and/or proteins have greatly facilitated experimental analyses of protein function in cells and organisms, particularly where protein depletion results in loss-of-fitness. One such approach involves targeted protein degradation, which was not previously reported for the African trypanosome, Trypanosoma brucei, an otherwise experimentally tractable parasite that causes often lethal diseases in humans and animals.

 The current study presents compelling data reporting adaptation of the deGradFP system for insect-form T. brucei; proof-of-principle is demonstrated by targeting a nuclear protein and a cytoplasmic protein. As the authors point out, the deGradFP approach relies upon the GFP-tagged protein retaining function and the VhhGFP4 nanobody being able to access the tag (meaning that the tag and the nanobody must be present in the same cellular compartment). This approach, as also noted, could be particularly powerful and effective for linking primary loss-of-function phenotypes to proteins with normally slow turnover, since protein degradation using deGradFP is rapid (detectable after 3 h), while knockdown by the widely used RNA interference approach can be relatively slow.

 I suggest some minor/simple edits to improve clarity:

1. Fig. 1C, 2A, 2B, 3: The error bars are unclear. Suggest increasing the line weight.
2. Fig. 1 legend: Replace “dashed dashed” with “dashed lines”.
3. First paragraph of results: Replace “177 bp locus” with “177 bp repeats on minichromosomes”.

Some further suggestions that the authors may want to consider:

1. It’s likely that the two example proteins are degraded in a proteasome-dependent manner, but this could be tested using a proteasome inhibitor such as bortezomib.
2. First paragraph of results: The authors could add some detail explaining how Tb927.5.700 was selected/prioritised as the source of the F-box to be fused to the nanobody.
3. Discussion: deGradFP may also effectively target ‘nuclear’ proteins, either before they reach the nucleus or by acting directly in the nucleus. Indeed, it remains unclear at this point whether the centrally located La NLS effectively directs NLS-deGradFP to the nucleus. Also, some proteins that function in both compartments may be effectively targeted by deGradFP.
4. Consider depositing key plasmids with a plasmid repository such as Addgene.

Is the rationale for developing the new method (or application) clearly explained?

Yes

Is the description of the method technically sound?

Yes

Are the conclusions about the method and its performance adequately supported by the findings presented in the article?

Yes

If any results are presented, are all the source data underlying the results available to ensure full reproducibility?

Yes

Are sufficient details provided to allow replication of the method development and its use by others?

Yes

Reviewer Expertise:

Parasite genetics.

I confirm that I have read this submission and believe that I have an appropriate level of expertise to confirm that it is of an acceptable scientific standard.

# Peer review: Reviewer response for version 1

**DOI:** 10.21956/wellcomeopenres.19908.r51224

**Published:** 2022-07-11

**Authors:** Christopher L. de Graffenried

**Affiliations:** 1 Department of Molecular Microbiology and Immunology, Brown University, Providence, RI, USA

This work reports the implementation of a degron approach for protein depletion in *T. brucei*. While *T. brucei* benefits from RNA interference, degron approaches that target the protein pool directly are likely to be valuable tools for rapid, selective, and more complete protein depletion. Ishii and Akiyoshi have identified a domain of a trypanosomatid-specific SCF E3 ubiquitin ligase complex (FBP75) that can target proteins for degradation, which is an important advance. They have fused this degron domain to a single-chain antibody that recognizes GFP, which when expressed using a tetracycline-inducible system targets FP-tagged proteins in cells, likely leading to their ubiquitination and subsequent degradation via the proteasome. Expression of the FBP75-Vhh fusion on its own does not appear to be toxic, which is essential for this method. The authors have shown degron-tag specific depletion of two proteins- the kinetochore protein KKT3, which is nuclear, and Sec31, which is a component of the COPII complex in the cytosol. They argue that the depletion of these proteins is more rapid than RNAi and provides more acute growth defects.

 Overall, this work provides the exciting prospect of a functional degron system for use in trypanosomatids, which could be extremely useful as a complement to RNAi in *T. brucei* and allow the conditional depletion of essential proteins in *Leishmania* and *T. cruzi*, which is a pressing issue. I have one significant concern that could be addressed by citing specific previous results:

1. As the authors mention, the FBP75 domain has just been identified as a potential degron and we do not currently know which Skp1 or cullins the domain interacts with - this specific pathway in T. brucei is complex. Can the authors show that the Vhh antibody on its own is not sufficient to disrupt the function of or degrade the YFP-tagged proteins? It appears that the original Cassinus paper has this data (Figure 2B). Considering that a new degron candidate is being employed here, the evidence that the Vhh on its own does not cause degradation in other systems should be specifically mentioned.

Smaller comments not necessary to address in this manuscript but would be important for testing the mechanism and generality of the approach:

1. Does the expression of the Vhh-FBP75 fusion lead to the appearance of higher MW bands corresponding to ubiquitinated versions of the YFP-tagged proteins?
2. Does blocking proteasomal activity via MG132 limit the loss of YFP signal? Are the proteins mislocalized, even if they are still present in the cell?
3. It seems possible that degron-mediated depletion of proteins could provide different phenotypes compared to RNAi. Since the method could actively target proteins that are already part of stable protein complexes, such as the KKTs, ubiquitination of these proteins could lead to the direct disruption of complexes. That might provide different phenotypes compared to RNAi. So, speed of depletion is just one aspect of the potential benefits of the degron. Have the authors observed any differences in how the phenotypes manifest?
4. The authors do not provide a side-by-side comparison of the Sec31 RNAi and Sec31 degron. The paper they cite for the Sec31 RNAi shows a slower growth defect than the degron, but it should be noted that at longer time points the RNAi is showing a very lethal phenotype while the degron is showing what appears to be a rebound in the population. This could be due to a loss of expression of the Vhh-degron, which can occur with tetracycline-inducible systems. This might be a persistent issue with this implementation of the degron approach.
5. Figure 1 legend: “Green dashed dashed” - Remove one of the “dashed.”
6. Figures 1 - 3: Error bars aren’t clear. I would recommend making them more visible by using a thicker line size.
7. Figures 2 and 3: The authors could zoom in to a smaller area for the fluorescence images so that we can see the YFP image in more detail.
8. To our knowledge, the CRISPR/Cas9 approach used/described in Beneke *et al.*, 2017 is not a conditional KO. It is described as such in the introduction.

Is the rationale for developing the new method (or application) clearly explained?

Yes

Is the description of the method technically sound?

Yes

Are the conclusions about the method and its performance adequately supported by the findings presented in the article?

Yes

If any results are presented, are all the source data underlying the results available to ensure full reproducibility?

Yes

Are sufficient details provided to allow replication of the method development and its use by others?

Yes

Reviewer Expertise:

parasitology, chemical biology, cytoskeleton

I confirm that I have read this submission and believe that I have an appropriate level of expertise to confirm that it is of an acceptable scientific standard.

# Peer review: Reviewer response for version 1

**DOI:** 10.21956/wellcomeopenres.19908.r51226

**Published:** 2022-06-29

**Authors:** Christine Clayton

**Affiliations:** 1 Heidelberg University Centre for Molecular Biology (ZMBH), Heidelberg, Germany

I will not summarize the article since the Abstract is perfectly adequate.

 This method should be extremely useful for studying the functions of proteins that are difficult to deplete by RNAi, or which show slow depletion so that it is difficult to distinguish between primary and secondary effects. The authors carefully highlight the possible drawbacks, especially not knowing whether it works in bloodstream forms, and target protein accessibility. It is likely that proteins that are in membrane-bound compartments (glycosome, mitochondrion, ER) will be less susceptible. In future, it would be interesting to know whether it was really necessary to express the nuclear-targeted version of the nanobody fusion in order to get degradation of the kinetochore protein, and what happens for proteins that shuttle between nucleus and cytoplasm. It's also possible that some "accessible" proteins will be less amenable than others, as happens with other degron systems - only testing will reveal this.

 It would have been useful to supply annotated versions of the plasmid sequences and some maps. I'm sure researchers could work this out but it would save time to supply the details in advance. On the other hand it's really nice that the sequences are already supplied, and the colour-coding on the sequence Table is already very helpful.

 I have a small quibble. The statement "kinetoplastids may represent one of the earliest-branching eukaryotes based on a number of unique molecular features" with the reference to Cavalier-Smith's paper in 2010 should probably be removed. Most of the arguments in that paper have since been invalidated. Cavalier-Smith's conclusion is based mainly on the absence of Tom40 and ORC complexes. Both Tom40 and ORC complexes are known to be present, although highly diverged from those of other model organisms. In contrast, for example, some Kinetoplastid protein sequences (e.g. Tom22) resemble plants more than yeast. Other remaining arguments for things that are missing or unusual are also out of date. Possibly more remarkable is the completely new kinetochore composition in trypanosomes, discovered by the current author. However, it depends on what you judge to be important - what about (for example) the novel proteins that replace histones in Dinoflagellates? Burki *et al.* (2019 ^1^), based on seqeunces, put all of the Excavata and various other things as branching separately, but on the same "level" as separation of Amorphea from Plants and SAR, with no way of seeing what came first. Trying to pick out a few organisms as being earliest-branching based on particular features is simply too subjective.

Is the rationale for developing the new method (or application) clearly explained?

Yes

Is the description of the method technically sound?

Yes

Are the conclusions about the method and its performance adequately supported by the findings presented in the article?

Yes

If any results are presented, are all the source data underlying the results available to ensure full reproducibility?

Yes

Are sufficient details provided to allow replication of the method development and its use by others?

Partly

Reviewer Expertise:

Trypanosome gene expression

I confirm that I have read this submission and believe that I have an appropriate level of expertise to confirm that it is of an acceptable scientific standard.

## References

[1] : The New Tree of Eukaryotes. *Trends Ecol Evol* .35(1) : 10.1016/j.tree.2019.08.008 43-55 10.1016/j.tree.2019.08.008 31606140

# Peer review: Reviewer response for version 1

**DOI:** 10.21956/wellcomeopenres.19908.r51223

**Published:** 2022-06-27

**Authors:** Cynthia He Y

**Affiliations:** 1 Department of Biological Sciences, Centre for BioImaging Sciences, National University of Singapore, Singapore, Singapore

A deGradFP system that allows inducible, targeted protein degradation in *T. brucei* is described. As a proof-of-concept, the system is successfully used to target the degradation of one nuclear protein and one cytoplasmic protein. This is the first targeted protein degradation method reported in *T. brucei*, expanding the current molecular toolkits available in this important model organism. Rapid protein degradation makes it very useful for protein functional studies.

 As the method relies on the physical recruitment of the ubiquitin ligase complex to the targeted protein through YFP binding and KKT3 is present in a protein complex, it may be worth addressing whether other components in the KKT complex could also be degraded through this process. Such information may help to understand any potential “off-target” effects of this method.

 Another minor suggestion is to make the error bars larger in all the growth curves. They are barely visible in the current figures.

Is the rationale for developing the new method (or application) clearly explained?

Yes

Is the description of the method technically sound?

Yes

Are the conclusions about the method and its performance adequately supported by the findings presented in the article?

Yes

If any results are presented, are all the source data underlying the results available to ensure full reproducibility?

Yes

Are sufficient details provided to allow replication of the method development and its use by others?

Yes

Reviewer Expertise:

T. brucei cell biology

I confirm that I have read this submission and believe that I have an appropriate level of expertise to confirm that it is of an acceptable scientific standard.
